# Morphological Analysis and Interaction of Chlorophyll and BSA

**DOI:** 10.1155/2014/872701

**Published:** 2014-05-18

**Authors:** Filipe D. S. Gorza, Graciela C. Pedro, Tarquin F. Trescher, Romário J. da Silva, Josmary R. Silva, Nara C. de Souza

**Affiliations:** Grupo de Materiais Nanoestruturados, Universidade Federal de Mato Grosso, 78600-000 Barra do Garças, MT, Brazil

## Abstract

Interactions between proteins and drugs, which can lead to formation of stable drug-protein complexes, have important implications on several processes related to human health. These interactions can affect, for instance, free concentration, biological activity, and metabolism of the drugs in the blood stream. Here, we report on the UV-Visible spectroscopic investigation on the interaction of bovine serum albumin (BSA) with chlorophyll (Chl) in aqueous solution under physiological conditions. Binding constants at different temperatures—obtained by using the Benesi-Hildebrand equation—were found to be of the same order of magnitude (~10^4^ M^−1^) indicating low affinity of Chl with BSA. We have found a hyperchromism, which suggested an interaction between BSA and Chl occurring through conformational changes of BSA caused by exposition of tryptophan to solvent. Films from BSA and Chl obtained at different Chl concentrations showed fractal structures, which were characterized by fractal dimension calculated from microscopic image analysis.

## 1. Introduction

Binding of human serum protein (HSA) with different compounds has been an intense research field in chemistry, biology, and medicine. Processes such as absorption, excretion, and toxicity of drugs, for instance, during chemotherapy [1]—can be affected by the binding of these compounds with HSA [2, 3]. Several investigations from experimental and theoretical [4–7] viewpoints on the binding of HSA with drugs have been performed. Some used drugs were 3′-azido-3′-deoxythymidine (AZT), aspirin, taxol, cisplatin, atrazine, 2,4-dichlorophenoxyacetic (2,4-D), polyamines, chlorophyll, chlorophyllin, poly(ethylene glycol), vanadyl cation, vanadate anion, cobalt-hexamine, and arsenic trioxide (As_2_O_3_), astilbin [8].

An alternative to the HSA is the bovine serum albumin (BSA), which has been used as a model of proteins for the study of biophysical and physicochemical processes. BSA is very attractive because it has low cost, stability, structural homology of 80% with HSA, water solubility, and versatile binding ability [9]. A lot of studies on interaction of BSA and ligands have been carried out. In these works, compounds including 2-(4-N,N-dimethylamino) phenylimidazo [4,5-b]pyridine (DMAPIP-b) [10], imidazolium chloride ionic liquids [11], prednisolone [12], aspirin [13], resveratrol [14], genistein [14], curcumin [14–16], malachite Green [17], bright red 6C [18], anticancer drugs [19], heparin [20], and ascorbic acid [21] were used. In particular, the behavior of the binding of chlorophyll (Chl) to BSA has been also examined [22] because it has been shown that this ligand can exhibit antimutagenic property against several potential human carcinogens [23, 24], action antioxidant [25], and antigenotoxic [26]. It is considered as a drug due to its bactericidal activity and high performance healing of wounds in addition to acting as an antioxidant in burns [27]. It has also been used as a photosensitizer for photodynamic therapy drug for its high absorptivity in the visible light region of the electromagnetic spectrum and low toxicity. Despite these studies, interaction of BSA and Chl by analysis of casting films from these compounds has not been carried out yet. Therefore, studying the interaction of Chl with BSA in aqueous solution and solid state (as films) can be used as a model for elucidating the Chl-HSA complex. Chl can bind to biological proteins modulating their activities. This bind process is determined by the behavior of interactions between drugs with proteins [21]. To the best of our knowledge, there have been no studies on interaction between Chl and proteins in casting films.

In this paper, we report on the study of interaction between Chl and BSA in aqueous solution by using UV-Vis spectroscopy, which allowed the determining of the binding constants at different temperatures. In addition, casting films were prepared from BSA and Chl in which—by using optical microscopy—the morphological structures found were studied.

## 2. Materials and Methods

Chlorophyll (MP Biomedicals) and BSA (fraction V, purity 96–100%) from Acros Organics were used as received. Chl stock solution was prepared by dissolving Chl in purified water with a concentration of 5.5 × 10^−4^ mol·L^−1^ (0.5 g·L^−1^). The pH of the Chl solution was adjusted to 8 (close to physiologic pH) by adding appropriate amounts of ammonium hydroxide. BSA stock solution was obtained by dissolving the BSA in PBS buffer, physiological pH, with a concentration of 7.6 × 10^−6^ mol·L^−1^ (0.5 g·L^−1^). Solutions at concentrations below were obtained from stock solutions. The films were prepared by casting 100 *μ*L of solutions on a quartz slice (36.0 mm × 14.0 mm × 1.0 mm). The adsorbed amount, which is proportional to absorbance, was monitored by measuring the UV-Vis absorption spectra with a double-beam Thermo Scientific spectrophotometer model GENESYS 10. Surface morphological structure was investigated by using an optical microscope (model Eclipse Ci/L, Nikon).* ImageJ* software [28] was employed to determine the fractal dimension using the box counting method.

## 3. Results and Discussion

### 3.1. Study of Solutions

**Figure 1:**
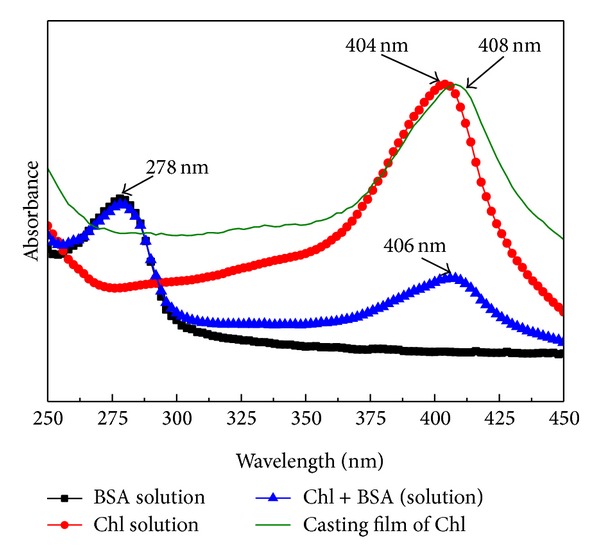
Spectra of BSA, Chl, BSA + Chl solutions, and Chl casting film at 23°C.

Figure 1 displays the spectra of BSA and Chl solutions as well as the spectra of the films and mixing of solutions. For BSA solution, it is possible to note a typical absorption peak at 278 nm, which is attributed to tryptophan amino acid and tyrosine [29]. In contrast to Chl in solution (404 nm), the absorption peak of Chl films appears at 408 nm. These results reveal a red shift of 4 nm for the film in relation to aqueous solution. According to the exciton theory, this shift indicates the occurrence of aggregation [30]. It is well known that when the dye molecules in the solution phase are transferred to a solid surface, molecular aggregation may occur [31].

**Figure 2:**
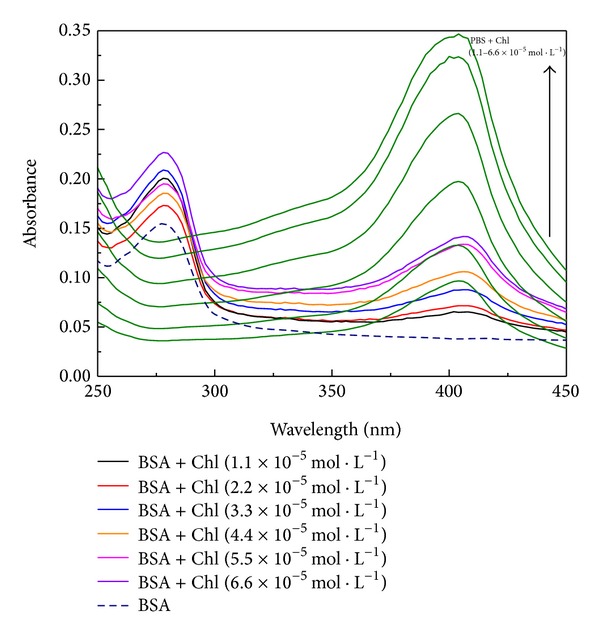
Spectra of BSA solution before (- - -) and after addition of Chl at different concentrations. The green spectra are the Chl at different concentrations in PBS solution without BSA.

The UV-Vis spectroscopy technique is a simple and effective method to investigate the molecular interaction and complex formation [21]. UV-Vis analyses were performed for BSA in solution with physiological pH and concentration of 1.9 × 10^−6^ mol·L^−1^ (required for better visualization of the band at 278 nm) and modified solution after the addition of Chl at different concentrations. All of these experiments were carried out using 2.0 mL of BSA solution contained in a quartz cuvette. The amount of Chl added was the same (40 *μ*L) for all concentrations examined (1.1, 2.2, 3.3, 4.4, 5.5, and 6.6 × 10^−5^ mol·L^−1^). The experiment was repeated in PBS solution without BSA (green curves in Figure 2). This procedure aims to discard the effect of absorption of Chl. The difference between the spectra with and without BSA reveals the behavior of the BSA front of Chl.

Figure 2 shows the UV-Vis spectra for BSA solution (dot curve) and BSA with aliquot of 40 *μ*L of Chl at different concentrations. The green spectra are aliquot of Chl at different concentration with PBS solution. Hyperchromism effect was observed with increasing Chl concentration in BSA. This effect can be associated with the interaction of BSA with Chl and may be indicative of an increase in exposure of tryptophan to the solvent [21, 32] due to a conformational change in the protein [33].

**Figure 3:**
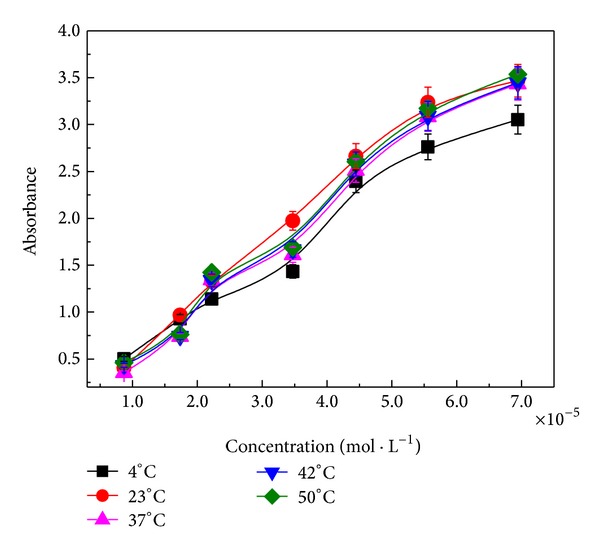
Absorbance versus concentration of Chl obtained at different temperatures. The curves were evaluated at 278 nm.

Figure 3 shows a plot of absorbance as a function of Chl concentration at different temperatures. No great differences are observed by changing the temperature, but it is noted that the absorbance increases with increasing Chl concentration. As discussed above, this hyperchromism may indicate an interaction between BSA and Chl [33–35].

### 3.2. Determination of Binding Constants

The determination of binding constant of Chl with BSA was determined by using a double reciprocal plot, which was obtained in terms of absorbance changes at 278 nm as a function of reciprocal concentration according to the Benesi-Hildebrand equation [16, 34]:

(1) $$\begin{array}{c} \frac{1}{\Delta A_{\text{abs}}}=\frac{1}{\left[S\right]\left[L\right]\varepsilon K}+\frac{1}{\left[S\right]\varepsilon }, \end{array}$$

where Δ*A*_abs_ is the change in the absorbance at 278 nm, *K* is the binding constant, [*S*] is the concentration of BSA, [*L*] is the concentration of Chl, and *ε* is the extinction coefficient.

**Figure 4:**
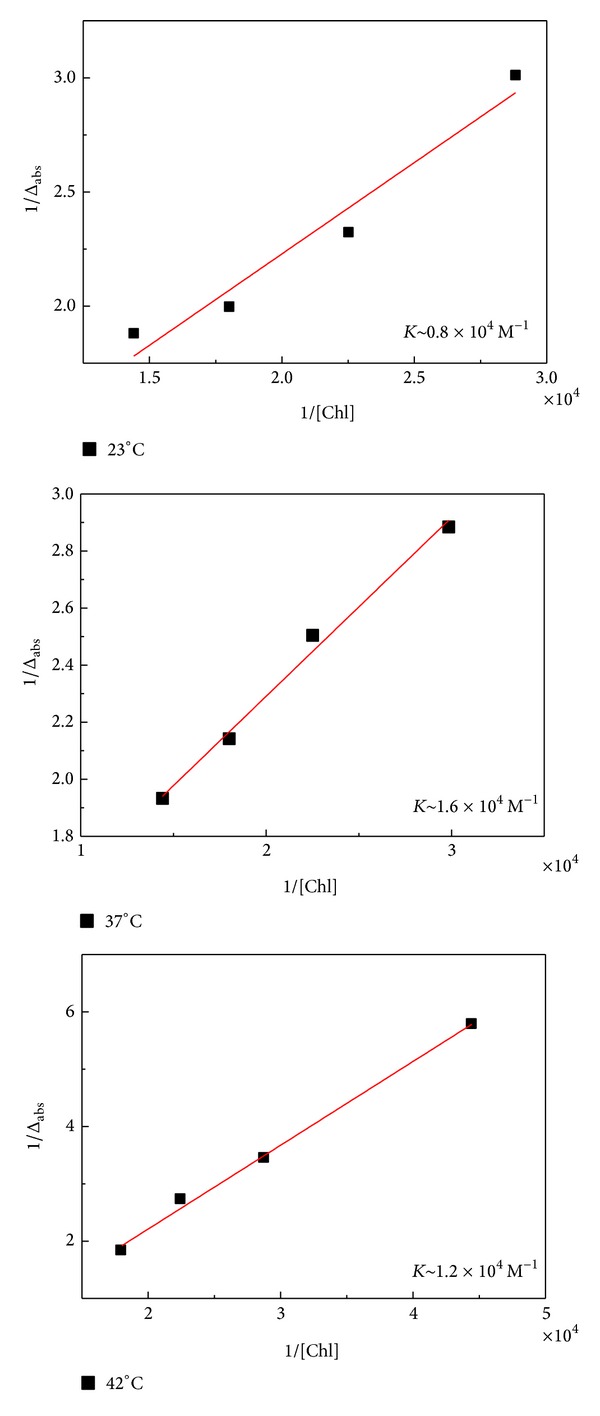
Linear plots for 1/Δ*A*_abs_ versus 1/[Chl] at 23, 37, and 42°C.

Figure 4 shows the linear plots of 1/Δ*A*_abs_ versus 1/[Chl] for aqueous solution of BSA and Chl at 23, 37, and 42°C. They were obtained by changing the Chl concentration as shown in Figures 2 and 3.

The changes in absorbance values were followed at 278 nm. The binding constants have been estimated to be *K* ~ 10^4^ M^−1^ for all the temperatures used. This value is similar to that found in literature for HSA-Chl complexes [2, 3]. Values of the binding constant in the range 1–15 × 10^4^ M^−1^ are considered moderate [36]. In this case, absorption and distribution of drugs for tissues are feasible. Then, our results suggest that even for temperatures of 23 and 42°C, which means hypothermia and fever for human body, the absorption and distribution of Chl for tissues are available.

### 3.3. Morphological Analysis

**Figure 5:**
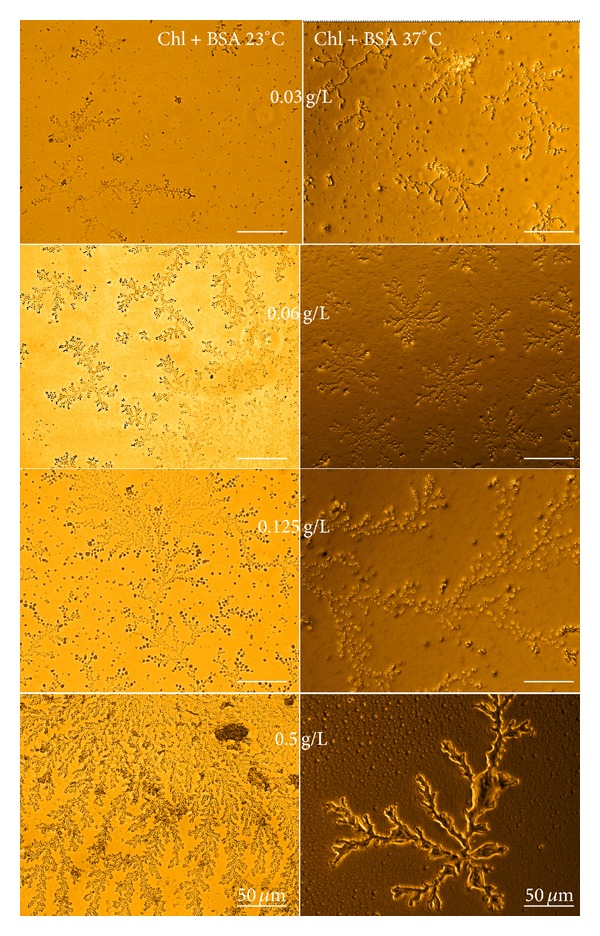
Optical microscopic images of films from BSA + Chl at 23 and 37°C at different Chl concentrations.

Figure 5 shows optical microscopic images for films from BSA + Chl at different concentrations of Chl. We note fractal structures for all of the Chl concentrations used. Films from pure BSA do not exhibit fractal structures, whereas those prepared from pure Chl exhibit several fractals (data not shown). Fractals have branched structures; however, larger branches are observed in films obtained at 37°C. The difference between the values of Df (fractal dimension) for casting films obtained at different temperatures can be related to the procedure and the time required for drying, since dry films at 37°C were prepared in an oven while others were obtained at room temperature.

In order to gain an insight in formation mechanism of the fractal structures, the concept of fractal dimension was used to characterize fractal structures [37, 38]. We have determined the fractal dimensions of the structures by using the box counting method [39].  Table 1 displays the fractal dimensions from Figure 5. It is noted that the fractal dimension increases as function of concentration. This arises from the increase in size of fractal structures. Special attention should be paid to *D*_*f*_ ~ 1.6, which was obtained from the fractal structures at concentration of 5.5 × 10^−4^  mol·L^−1^. This value is close to *D*_*f*_ = 1.7, predicted for the diffusion-limited aggregation (DLA) model [39, 40]. This result is consistent with the aggregation suggested by the finds using UV-Vis spectroscopy (Figure 1) and suggests the complex formation of BSA with Chl.

## 4. Conclusion

We have investigated the interaction of BSA with Chl in aqueous solution as well as the morphological structures formed in films from these two compounds. Albumins promote the transport of material in the blood and are able to bind with different biologically active compounds, such as drugs, steroids, and dyes. As only free drug can exert their action, some factors influence their effectiveness in the active site. One such factor is the affinity of the protein drug which can be measured by the binding constant between them. The binding constant was found to be stable around 10^4^ M^−1^ for all temperatures employed. This indicates a moderate affinity between BSA and Chl, which is suitable to absorption and transportation of this compound. It revealed a hyperchromism indicating an interaction of BSA and Chl, which can occur through conformational change of BSA caused by exposition of tryptophan to solvent. Films from BSA show that fractal dimension depends on concentration which is associated with the increase of size of the structures. At concentration of 5.5 × 10^−4^ mol·L^−1^, the fractal dimension is found to be *D*_*f*_ ~ 1.6, which is close to *D*_*f*_ = 1.7 predicted for the diffusion-limited aggregation (DLA) model. Alternative methods such as microscopy and fractal analysis may lead to relevant insights into the interactions which occur between HSA with Chl in human body.

## Figures and Tables

**Table 1:** Fractal dimension values for the films prepared from BSA and Chl at 23 and 37°C at different concentrations.

Conc.	Chl + BSA	Chl + BSA
Chl (mol·L^−1^)	23°C	37°C
3.3 × 10^−5^	1.25	1.31
6.6 × 10^−5^	1.27	1.33
1.4 × 10^−4^	1.37	1.50
5.5 × 10^−4^	1.65	1.62
